# Procedure-level data linkage to drive improvement in case ascertainment for the Australian Breast Device Registry

**DOI:** 10.1177/18333583251352621

**Published:** 2025-07-16

**Authors:** Dilinie Herbert, Saeid Kalbasi, Natalie Heriot, Delphine Allan, Patrick Garduce, Ahmad Reza Pourghaderi, Sally McInnes, Susannah Ahern

**Affiliations:** Monash University, Australia

**Keywords:** registry, breast implant, data linkage, health information management, device monitoring

## Abstract

**Background:** The Australian Breast Device Registry (ABDR) monitors breast device safety by collecting procedure data from clinicians across Australian jurisdictions. Ensuring high case ascertainment, including implant insertion and revision, is essential. By linking with an administrative dataset, the ABDR can identify hospital and procedure-level data gaps to assess case ascertainment more effectively, supplementing previous efforts using breast device sales data. The aim of this project was to link ABDR data with the Victorian Admitted Episodes Dataset (VAED) to determine total and procedure-level case ascertainment and to provide feedback to participating sites regarding their data capture to support quality improvement. **Method:** The ABDR applied to the Centre for Victorian Data Linkage (CVDL) to administer the data linkage, employing a series of Australian Classification of Health Intervention (ACHI) procedure codes. Then, using this data, the ABDR produced site-specific case ascertainment reports. **Results:** The CVDL was able to match ABDR breast device-related procedures to the VAED dataset, demonstrating an overall 79% case ascertainment. Tissue expander removal and implant insertion procedures were most commonly captured (89%) and those least captured were tissue expander revision and removal or replacement procedures (59%). Customised site-specific reports were developed and distributed, comprising a series of benchmarked line graphs to track site procedure ascertainment over 6 years. **Conclusion:** Data linkage informed ABDR total and procedure-level case ascertainment in Victorian public and private hospitals. Reporting back to hospitals, their individual case ascertainment is integral to addressing gaps in case reporting and improving overall registry data capture and completeness. The registry proposes to complete data linkage annually in Victoria to monitor improvements in case reporting, and explore using data linkage in other health jurisdictions in the future. **Implications for health information management practice:** Data completeness is critical to data quality and use for clinical decision-making. Third-party data verification processes are a useful activity to enhance the quality of health service-contributed data to registries.

## Introduction

Clinical quality registries (CQRs) are valuable tools that, when mature and supported by the clinical community, can facilitate significant improvements to the quality and safety of healthcare and patient outcomes (Evans et al., 2011; Hoque et al., 2018). While registry science is a relatively new field in Australia, the Australian Commission on Safety and Quality in Health Care (ACSQHC) has long recognised registries’ value in monitoring and reporting health information on a national scale (ACSQHC, 2024). CQRs collect longitudinal data that is analysed to determine emerging trends as well as monitor performance against, and adherence to, clinical benchmarks. Registry impact is greatest when population capture is high. Wilcox and McNeil (2016) recognised that high capture of the eligible population is “critically important” to ensure that selection bias is minimised. However, this is often difficult because of missing data (Thygesen and Ersbøll, 2014).

The Australian Breast Device Registry (ABDR) is a CQR established in 2014 that captures longitudinal health outcomes and emerging trends relating to breast device surgery (Cooter et al., 2015). It is funded by the Australian Government Department of Health, Disability and Ageing under its National CQR Program, and managed by Australia’s largest manager of clinical registries, Monash University’s School of Public Health and Preventive Medicine. It is endorsed by the three prominent specialist medical colleges in the field: the Australian Society of Plastic Surgeons, the Australasian College of Cosmetic Surgery and Medicine and Breast Surgeons of Australia and New Zealand.

The ABDR monitors the long-term safety and performance of breast devices, identifying and reporting on trends, complications and the quality of surgery involving breast implants, tissue expanders, acellular dermal matrices and other mesh, including procedures involving the insertion, revision or removal of breast devices. As with many registries, the ABDR employs an opt-out consent model that is associated with greater population capture (McNeil et al., 2010). While registry participation is not mandatory for either patients or clinicians/surgeons, all healthcare facilities where breast device procedures are performed are eligible to participate in the registry. The ABDR currently has 239 participating public and private health services across all jurisdictions, with the exception of Western Australian public hospitals, where state legislation prohibits the use of the opt-out model (Ahern et al., 2024).

Despite nearly a decade of operation, the registry has encountered persistent challenges in calculating a complete and current denominator comprising all hospital sites and clinicians involved in breast device surgery. This difficulty arises from the dynamic nature of healthcare providers and facilities, with frequent changes in clinical staff and institutional participation, leading to changing denominators. Maintaining up-to-date information in real time thus presents significant logistical obstacles. Traditionally, the ABDR has employed several strategies to improve case ascertainment through recruitment activities, including engaging clinicians at professional conferences, reaching out to new college fellows with the assistance of the specialist medical colleges, and monitoring the websites of participating hospital sites. These methods allow the registry to identify changes in clinical practice or the addition of new clinicians; however, they offer only a partial solution to the problem of maintaining comprehensive and accurate records.

The ABDR actively sought to approximate case ascertainment using other strategies. It has utilised publicly available data from the Australian Institute of Health and Welfare (AIHW); however, these data are typically organised by financial year rather than a calendar year and lag real-time data by approximately 12 months. This time discrepancy limits its effectiveness for real-time case ascertainment but still serves as a useful supplementary tool for approximating registry data completeness. Over the last 3 years the registry has partnered with the Therapeutic Goods Administration (TGA) to receive de-identified breast device sales data. Breast device sales data are reported by industry to the TGA, which subsequently shares high-level data with the ABDR to support case ascertainment of breast device insertions. This approach differs from procedure-level case ascertainment, as not all procedures involve the insertion of a new device, such as cases of device explanation or malposition procedures. This analysis suggested that on average the registry was capturing 70% of all breast devices reported (although this varied, for example it was 86% in 2023), similar to the proportion capture rate using the AIHW data (72% across 2012–2023; Ahern et al., 2024). However, this is likely an underestimation of the true data capture, as breast device sales data include devices purchased but not used within the reporting period. Therefore, the registry decided to investigate administrative data linkage at a state level as a useful measure of case ascertainment because it provides data completeness (case ascertainment) at the hospital/site level.

Most health jurisdictions have infrastructure for conducting data linkage services with their administrative datasets. The Centre for Victorian Data Linkage (CVDL) is based within the eHealth Division of the Victorian Government Department of Health. The CVDL is approved to perform linkages with administrative health datasets, including the Victorian Admitted Episodes Dataset (VAED), the Victorian Emergency Minimum Dataset and the Elective Surgery Information System. In Victoria, mandatory reporting of inpatient data is captured in the VAED (Heslop et al., 2004) and was therefore selected as an appropriate administrative dataset against which the ABDR could assess its current case ascertainment rate within Victoria. In addition, CVDL has access to both public and private health data, and given that the vast majority of breast device procedures occur in private hospitals (Ahern et al., 2024), it was determined that Victoria would be an appropriate jurisdiction to trial this project.

The aim of this project was to link ABDR data to VAED records to quantify total and procedure-level case ascertainment of Victorian hospitals participating in the ABDR. The expectation was that by reporting this back to individual Victorian hospitals to assist them in auditing their procedure capture rates, this information would improve case ascertainment over time. Clinicians and site staff could then address any deficiencies in processes to collect and report their procedures to the ABDR and improve their data reporting, leading to higher overall ABDR case ascertainment.

## Method

### Population

The ABDR is a population-based registry collecting device and surgical details relating to cosmetic and reconstructive breast device procedures. In 2023, the total accrued data in the registry related to 98,460 patients, 113,439 procedures and 192,706 devices (Ahern et al., 2024).

### Data collection and selection

A data linkage application was originally submitted to CVDL in 2021. Due to delays associated with determining the optimal method of data linkage between the two organisations, the CVDL subsequently approved access to data extending through to 2022. Therefore, cases reported to the ABDR between 1 January 2017 and 31 December 2022 were utilised in this data linkage. A Monash University-hosted secure drive was selected as the secure data transfer platform. Data linkage was completed and results were reported back to the ABDR in October 2023.

To conduct case ascertainment, procedures in the VAED dataset that involved insertions, replacements, removals, repositions of breast implants and tissue expanders had to be identified and selected. The Australian Classification of Health Interventions (ACHIs) is the national standard for intervention coding in Australia. Ten ACHI procedure codes, selected according to three Block Numbers, were mapped to ABDR procedure types. The ABDR team conducted the mapping process with guidance from the clinical leads (representing each of the specialist medical colleges) and clinical coders, whose expertise was instrumental in aligning the ABDR operation types with the corresponding ACHI codes and descriptions, thereby ensuring accurate and consistent mapping. The ABDR procedure types associated with the ACHI codes were: first implant insertion; tissue expander insertion; tissue expander revision, removal or replacement; tissue expander removal and implant insertion; implant revision, removal or replacement; and implant removal and tissue implant insertion (Box 1).

**Box 1.**
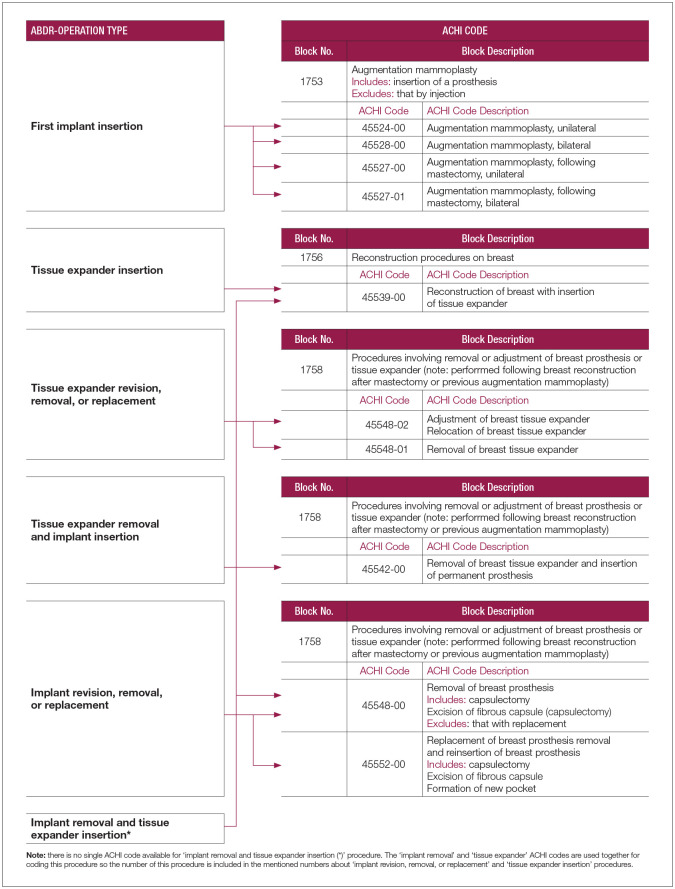
Mapping of ABDR operation types to ACHI procedure codes. *Source*: The Australian Breast Device Registry Annual Report 2023. Ahern et al. (2024), p. 22 School of Public Health and Preventive Medicine, Monash University. ABDR: Australian breast device registry; ACHI: Australian classification of health interventions.

### Data linkage procedure

The personal identifiers used in this data linkage were: name, date of birth, gender, Medicare number, unit record number (UR number), address, operation date, hospital name and hospital type. A data extract containing registrant and procedure information was generated based on the personal identifiers. The CVDL matched these cases against the VAED. The results were cross-referenced to verify the absence of errors.

### Reporting

The ABDR utilised the linked dataset to determine Victoria-wide case ascertainment for each of the years 2017 to 2022, in breast device procedures as a whole and for specific types of procedures. This information was then used to develop site-specific benchmarked reports. These reports compared the number of cases submitted by each site to the ABDR against the number of corresponding cases recorded in the administrative dataset (VAED). This approach allowed for a comprehensive assessment of data reporting completeness and discrepancies across sites within Victoria. Individual hospital reports were prepared and distributed in October 2024.

### Ethics approval

Data linkage activities are covered under the Human Research Ethics Committee approval for the ABDR (HREC number: 5/15).

## Results

The VAED records indicated that during the period of linkage, there were 347 hospitals in Victoria, each with a unique site identification code. There was, however, some discrepancy in the naming convention. The ABDR uses the List of Declared Hospitals (published by the Australian Government Department of Health and Aged Care) to record hospital names, in some cases the name recorded in the ABDR did not match the VAED. Therefore, the VAED list had to be cross-referenced to match hospital names recorded by the ABDR before the data linkage, which took some months. The ABDR confirmed that 74 of those hospitals contributed data to the registry (representing all Victorian public hospitals; Table 1, row 1).

**Table 1.** Overall proportion of Victorian public and private hospital sites matched (2017–2022).

Dataset	Public sites	Private sites
ABDR	24	50
VAED	41	58
% Sites captured by the ABDR	59%	86%

ABDR: Australian breast device registry; VAED; Victorian admitted episodes dataset.

### Total and procedure-level case ascertainment

The CVDL compared the ABDR Victorian data with VAED data for public and private hospitals between 2017 and 2022. During this period, the ABDR collected data from 59% of Victorian public hospitals and 86% of Victorian private hospitals that undertook breast device procedures according to VAED data (Table 1). This equated to an average of 64% of all Victorian public site procedures, and 76% of all Victorian private hospital procedures being captured by the ABDR. Significant variation in completeness was reported at the procedure level. Procedures with the highest completeness were tissue expander removal and implant insertion (89%), and tissue expander insertion (86%) – both procedures undertaken generally for reconstructive surgery. The procedure with the lowest completeness was tissue expander revision, removal or replacement (69%). Breast implant insertions and revision/removal or replacements were captured at 79% and 78% respectively. Given that breast implant procedures had a much higher volume than tissue expander procedures, the average rate of case ascertainment overall was 79% (Table 2).

**Table 2.** ABDR case ascertainment at breast level by procedure type (2017–2022).

Procedure type	ABDR	VAED	Capture rate (%)
First implant insertion	20,494	25,905	79
Tissue expander insertion	1567	1832	86
Tissue expander removal and implant insertion	1462	1640	89
Implant revision, removal or replacement	10,309	13,276	78
Tissue expander revision, removal or replacement	192	327	59
Overall	34,024	42,980	79

ABDR: Australian breast device registry; VAED; Victorian admitted episodes dataset.

### Customised site-specific case ascertainment reports

**Figure 1.**
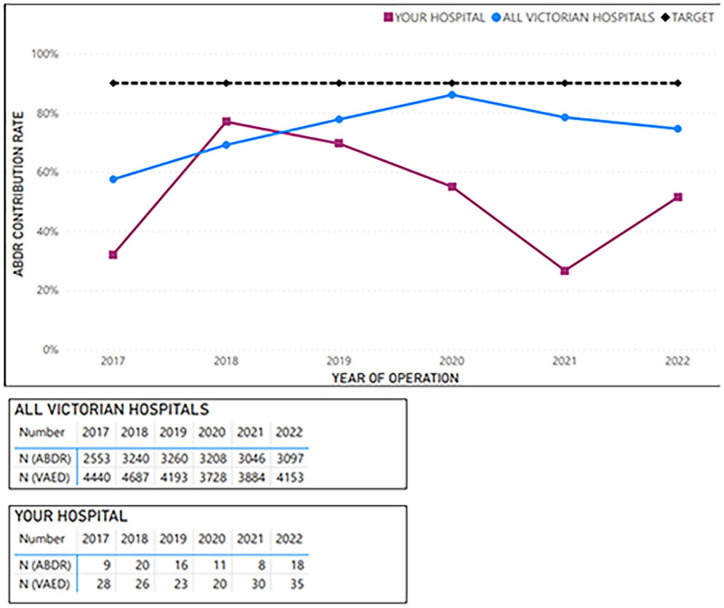
Example average sample hospital case ascertainment, benchmarked against Victorian average and target of 90% capture – total procedures (2017–2022).

**Figure 2.**
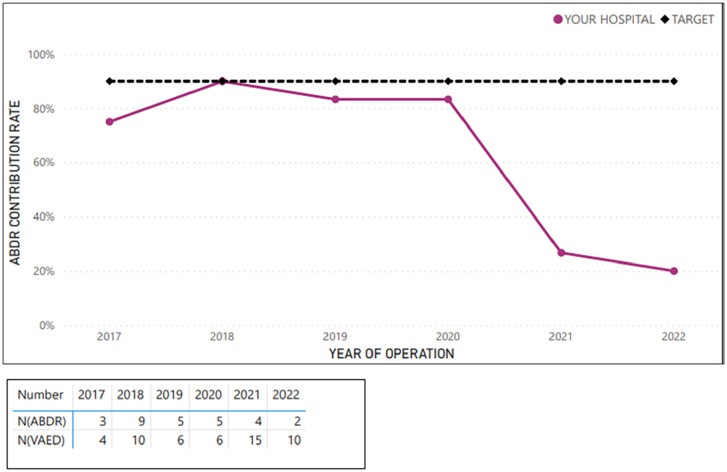
Example average sample hospital case ascertainment – first implant insertion procedures (at breast level; 2017–2022).

**Figure 3.**
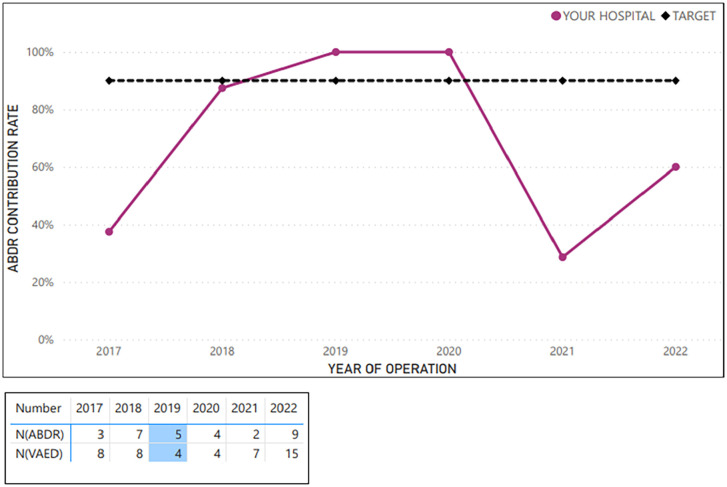
Example average sample hospital case ascertainment – tissue expander insertion procedures (at breast level; 2017–2022).

**Figure 4.**
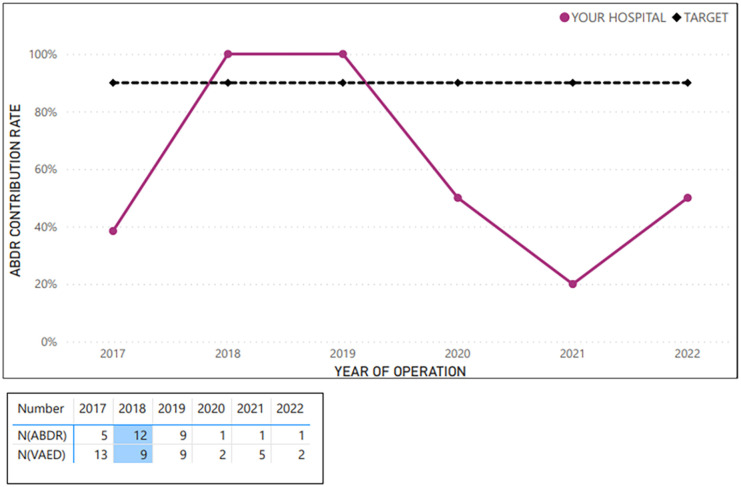
Example average sample hospital case ascertainment – tissue expander removal and implant insertion procedures (at breast level; 2017–2022).

**Figure 5.**
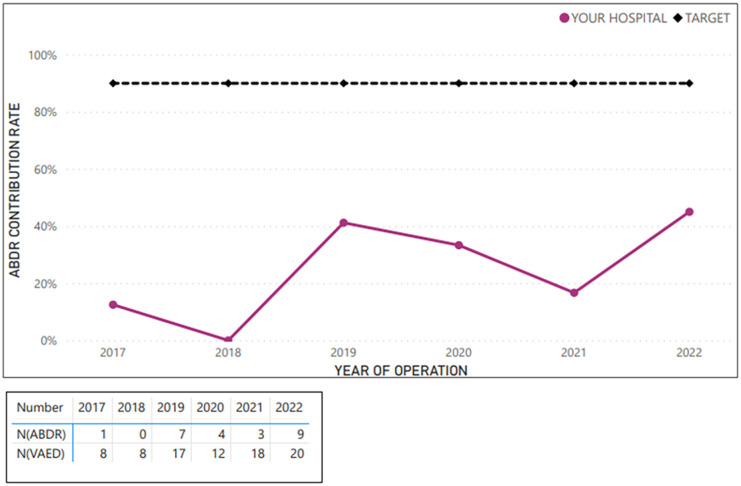
Example average sample hospital case ascertainment – implant revision, removal or replacement (at breast level; 2017–2022).

**Figure 6.**
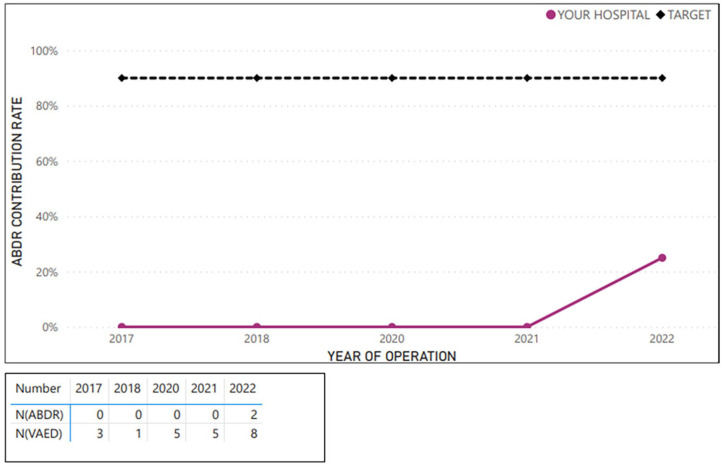
Example average sample hospital case ascertainment – tissue expander revision, removal or replacement (at breast level; 2017–2022).

The customised reports for each Victorian health service comprised six figures (e.g. shown in Figures 1–6). Figure 1 shows the average hospital case ascertainment over a 6-year period (red line), benchmarked against the Victorian average (blue line) and the target of 90% capture (black line). In Figure 1, the blue line represents the contribution rate for all sites by year. The average contribution rate (defined as procedures captured by the registry) was calculated by determining the ratio of breast device procedures captured by the VAED across all hospitals to the number of breast device procedures captured by the ABDR for each year. The red line reflects the contribution rate for that site. This calculation is repeated annually. Lastly, the black line indicates an aspirational benchmark of 90%. At present, no international benchmarks are available for case ascertainment within breast device registries. The Australia and New Zealand Bariatric Surgery Registry, which is comparable to the ABDR in both size and duration of operation, currently achieves a case capture rate of just over 80% (The Bariatric Surgery Registry Annual Report, 2023). In the absence of established benchmarks, the decision to adopt a 90% benchmark for the ABDR was informed by the desire to set an attainable target, reflecting a commitment to improve case ascertainment. This approach acknowledges the inherent complexities of case ascertainment while striving to optimise registry accuracy and coverage.

Figures 2–6 show the average hospital case ascertainment at a procedure level benchmarked against the target of 90%. It shows a hospital’s average contribution rate by procedure type. Here, there are two lines, the black 90% benchmark and the red line that reflect the average contribution rate of that procedure type to the registry. The ABDR was also able to show sites (in the accompanying tables) the number of procedures that were captured in the registry compared to those captured in the VAED. The light blue shading is used to show where the registry has captured more of that procedure than what is reported to the VAED (Figures 3 and 4).

## Discussion

Data linkage with mandatory-reported administrative health datasets can provide substantial value in quantifying registry case ascertainment, which is essential for achieving the meaningful impact of a CQR. This study identified variations in case ascertainment at both the hospital and procedural levels. To address these challenges and enhance case ascertainment, the ABDR developed customised, site-specific reports that are benchmarked against peer performance and an aspirational target. This approach aims to assist hospitals in identifying underreported procedures and supports ongoing efforts to improve case ascertainment over time.

High data quality and completeness are essential in registry sciences as they directly impact the validity and reliability of the findings derived from the dataset. Accurate and complete data ensure that analyses truly reflect real-world outcomes and enable robust risk modelling (Stey et al., 2015). Data missingness or inaccurate information can introduce bias, limiting the generalisability of results (Williamson et al., 2004), and hindering comparisons across different sites or populations. Furthermore, high-quality data enhance the registry’s utility for monitoring trends and evaluating device performance on an international scale (Cooter et al., 2015). Ultimately, comprehensive datasets ensure that registries fulfil their purpose of reporting health information on a national scale and improving patient outcomes (ACSQHC, 2024).

The data linkage with VAED offered valuable insight into where there are gaps in reporting to the ABDR. It is from the total case ascertainment that the registry can focus its attention to target and engage hospital sites. The procedural-specific data allows the registry the opportunity to provide training and education to sites to submit underreported procedures. The data linkage demonstrated that among public and private hospitals in Victoria, the sites not contributing to the registry account for a notable proportion of procedures not captured by the registry.

With respect to procedure-level case ascertainment, device insertion procedures are generally well captured (first implant insertion, tissue expander insertion and tissue expander removal and implant insertion). The discrepancy in procedure-level case ascertainment may be attributed to the current data collection process, wherein clinicians and their support staff manually record data using paper-based forms and subsequently submit these to the ABDR. As the ABDR relies on voluntary data collection by hospital staff, inconsistencies in data submission may arise. The registry anticipates that the customised site reports will highlight the variation in reporting over time, offering clinicians and support staff context to review work processes that can lead to improved reporting amongst underreported procedures. The registry can also be engaged in these efforts by scheduling in-service and training days with site staff. Addressing these gaps is essential to ensure that ABDR reports provide a more accurate representation of breast device procedures and outcomes. High data completeness in clinical registries is important to ensure that the quality improvement advice that the registry offers is based on valid data (McNeil et al., 2010). The ABDR is well positioned with its long history and support from specialist medical colleges to continue to improve its performance in this area.

Data linkage methods have been employed in Australian registries to enhance data accuracy and improve the assessment of clinical outcomes (Eliakundu et al., 2022; Kilkenny et al., 2019). Similar to the findings reported by Palamuthusingam et al. (2023), where variable agreement was observed between registry data and hospital data, our study identified discrepancies largely stemming from underreporting of particular procedure types. As a device registry, our primary focus remains on enhancing case ascertainment to improve capture rates, thereby ensuring more accurate and reliable data analysis and reporting. Reporting case ascertainment to hospitals illustrates any discrepancies between their clinically reported and administrative data, datasets that have two very distinct purposes. Clinical data are used for patient care, whereas administrative data are used to monitor service utilisation and payment. The customised site-specific reports generated by the ABDR offer a clear overview of data submission accuracy. Strategies the registry could offer sites to improve case ascertainment include workflow adjustments, regular audits or targeted training for hospital staff. The intention is to generate these reports annually to track progress and highlight where improvements in reporting have been made. By revealing discrepancies between the registry-reported data and administrative data, these reports are expected to prompt discussions amongst clinicians and site staff around registry contributions, and how they may implement ways to eventually improve case ascertainment and the reliability of the registry data.

### Limitations

A notable limitation of data linkage is that there are often times when cases are un-linked (or not matched), which can lead to biased results (Bohensky et al., 2010). Of note in this study is that there is not a precise match between ABDR operation type to ACHI code, and there might be errors in assigning ACHI codes to procedures at sites. Australia does not currently have a mandatory national unique health identifier. Although the Individual Healthcare Identifier is becoming increasingly used in healthcare delivery, it is currently variable. Therefore, it is possible for patient details to be incomplete or inaccurate between healthcare systems, which can lead to un-linked registry data to administrative data. The interpretation of the data linkage needs to be appreciated in light of these limitations. Factor analysis to compare the results of the linked and un-linked cases to determine any further significant differences was beyond the scope of the current study. Future research to systematically compare linked and non-linked cases will help to better understand the factors associated with case ascertainment.

## Conclusion

Data linkage with the VAED was a valuable opportunity to more accurately calculate ABDR case ascertainment. It was a formative activity that showed in total and at a procedure level, which breast device surgeries are reported to the registry and those that are commonly underreported. The customised site-specific reports are a way to communicate this information back to clinical leaders in order to improve case ascertainment. The ABDR will produce these reports annually with the aim of seeing improvements in case ascertainment over time. A similar process will be adopted for other health jurisdictions in the coming years.
